# Association between Prior *Chlamydia trachomatis* Infection and Ectopic Pregnancy at a Tertiary Care Hospital in South Western Uganda

**DOI:** 10.1155/2018/4827353

**Published:** 2018-03-01

**Authors:** Derrick Paul Mpiima, George Wasswa Salongo, Henry Lugobe, Augustine Ssemujju, Olivier Mumbere Mulisya, Abraham Masinda, Hillary Twizerimana, Joseph Ngonzi

**Affiliations:** Mbarara University of Science and Technology, Mbarara, Uganda

## Abstract

**Background:**

Increase in the number of ectopic pregnancy is attributed to increase in the incidence of pelvic infections. *Chlamydia trachomatis* is responsible for most of the sexually transmitted bacterial infections. If undetected and untreated, the infection can ascend to the upper genital tract and cause pelvic inflammatory disease (PID) and related sequelae (ectopic pregnancy and tubal factor infertility).

**Objective:**

To determine the association between prior *Chlamydia trachomatis* infection and ectopic pregnancy at Mbarara Regional Referral Hospital (MRRH).

**Methods:**

This was an unmatched case-control study carried out at MRRH involving 25 cases and 76 controls. Serological evidence of prior chlamydial infection was determined by testing for the presence of *Chlamydia* immunoglobulin G antibodies in their blood. Logistic regression was used to determine the association between prior *Chlamydia trachomatis* infection and also the factors associated with ectopic pregnancy. The significant level of <0.05 was used. *Results. Chlamydia* antibodies were found in 60% of patients with ectopic pregnancy and 26.3% of the controls (*p*=0.002). The presence of *Chlamydia* antibodies was associated with a fourfold risk of ectopic pregnancy.

**Conclusion:**

There was a strong association between prior *Chlamydia trachomatis* infection and ectopic pregnancy.

## 1. Introduction

In Uganda, in the last three decades, ectopic pregnancy has assumed epidemic proportion with significant maternal morbidity, mortality, and fetal wastage (Aliya (2010)). The incidence of ectopic pregnancy is directly related to the prevalence of salpingitis. Endosalpingeal damage secondary to pelvic inflammatory disease caused by *Chlamydia trachomatis* infection has been found to be a major risk factor for the development of ectopic pregnancy [1]. Studies from northern and southwestern Nigeria showed macroscopic evidence of pelvic inflammatory disease at laparotomy in over 60% of patients with ectopic pregnancy [2]. Both increased incidence of *C. trachomatis* infection and the efficacy of antibiotic therapy in preventing total tubal occlusion, after an episode of salpingitis, are related to increased incidence of ectopic pregnancy [3].

In females, up to 40% of chlamydial cervicitis may ascend to the endometrium and is responsible for the etiology of endometritis and salpingitis [4]. Fallopian tube samples of patients with ectopic pregnancy have also been found positive for *C. trachomatis* deoxyribonucleic acid using the polymerase chain reaction. The frequent association between chlamydial cervicitis and the presence of vaginal clue cells or Gram stain abnormalities indicative of an overgrowth of anaerobic bacteria has led to the speculation that *C. trachomatis* alters the normal vaginal ecology, thereby setting the stage for a complex polymicrobial upper genital tract infection.

Untreated or poorly treated chlamydial infection of the genital tract can therefore have serious long-term reproductive consequences. Late sequelae of the disease include chronic pelvic inflammatory disease, tubal blockage and infertility, chronic pelvic pain, and ectopic pregnancy [5, 6]. Seroepidemiological studies have indicated that chlamydial infections account for a large proportion of asymptomatic genital tract infection, by demonstrating a strong link between tubal pathology and the presence of chlamydial antibodies. The chlamydial immunoglobulin G antibodies are associated with the development of late sequelae and are markers for previous exposure. In chronically infected patients negative for endocervical *C. trachomatis*, a positive serological test may be the only indication of chlamydial involvement. Serum immunoglobulin G antibodies to *Chlamydia* have also been associated with tubal factor infertility and ectopic pregnancy [7].

This study therefore evaluates prior exposure to *C. trachomatis* in patients treated for ectopic pregnancy by determining the presence of immunoglobulin G antibodies in their sera. By comparing the same with women carrying normal intrauterine pregnancy, it seeks to determine the risk association between prior *C. trachomatis* infection and ectopic pregnancy at Mbarara Regional Referral Hospital.

## 2. Materials and Methods

This was an unmatched case-control study that is (1  :  3 ratio of cases to controls) carried out at Mbarara Regional Referral Hospital between September 2016 and January 2017. Consecutive sampling method was used to enrol all mothers who met the inclusion criteria until the sample size was achieved. The cases were 25 consecutive women with a diagnosis of ectopic pregnancy during the study period. The control group was made up of women with confirmed uncomplicated intrauterine pregnancy, attending the antenatal clinic of MRRH. Each case of ectopic pregnancy was followed by pregnant controls attending the antenatal clinic. The sample size for this study was calculated using the formula provided in Kelsey et al. (1996) for calculating the sample size for unmatched case-control studies. After computing for the expected 10% attrition, the sample size was calculated to be 25 cases and 76 controls giving a total sample size of 102. All patients who had laparotomy for tubal ectopic pregnancy, who consented to this study and satisfied the inclusion and exclusion criteria, were recruited until the minimum sample size was obtained. The approval to conduct the study was obtained from the Department of Obstetrics and Gynecology, Faculty of Medicine Research Committee (DMS 6), MUST Research Ethics Committee (Number 21/7-16), and the Uganda National Council of Science and Technology (HS 2146). Informed consent was obtained from all respondents, and confidentiality was ensured. Study participants were identified by study codes and not their names, for issues of confidentiality. In addition, authority was sought from the office of the hospital director of MRRH to conduct the study in this institution.

Serological evidence of prior chlamydial infection was determined in both groups by examining for the presence of *Chlamydia* immunoglobulin G antibodies in their blood. Five milliliters (5 ml) of blood was collected from the volar surface of the forearm of all the participants and emptied into clean, gold coated vacutainer. The specimen was collected from patients with ectopic pregnancy before blood transfusion and surgery and also from the control group. The blood was taken to the research laboratory (lancet laboratory) where the specimen was allowed to clot, centrifuged, and sera obtained. The sera were analyzed in batches for *Chlamydia* antibodies. The serological assay was done using the ImmunoComb *Chlamydia trachomatis* Immunoglobulin G kit (Orgenics, France), an indirect solid-phase enzyme immunoassay (EIA) test that quantitatively measures antibodies to *Chlamydia trachomatis* in human serum. The reagent test kit was brought to room temperature, and then, 10 *μ*L (microlitre) pipetted serum was assayed with reagent control samples for *Chlamydia trachomatis* antibodies.

The collected data were entered into a password protected computer using Microsoft excel, and data were imported into STATA 11 software for analysis. Results were presented as means with standard deviations, frequency distribution tables, and cross tables. Descriptive analysis was done for sociodemographic, sexual, and reproductive characteristic data, and tests of significance were done using chi-square test. The level of statistical significance was set at *p* value < 0.05. The regression logistic analysis was used to determine the association between *Chlamydia trachomatis* and ectopic pregnancy and also found the other factors associated with ectopic pregnancy.

## 3. Results

Majority of the participants were Banyankole, had at least primary level of education, and were residents of Mbarara. Mean age of the cases was 27.8 ± 4.3 and 25.2 ± 5.9 for controls. There were significantly more (12 (48.0%)) single women among the cases population with ectopic pregnancy than the controls (5 (6.58%)) (*p* < 0.01) (Table 1).

Table 2 shows sexual behaviour of the participants. The cases engaged in sexual intercourse at a significantly younger age than the controls (*p* < 0.01). There were more cases with multiple sexual partners compared to the control group. Previous history of pelvic inflammatory disease was obtained in 24 (96.00%) among the cases. This was significantly more than 8 (10.53%) among the controls (*p*=0.02). The cases group had significantly more surgeries 13 (52.00%) compared to only 6 (7.89%) among the control group.

*Chlamydia* antibodies were found in 15 (60%) and 20 (26.32%) of the cases and controls, respectively (*p*=0.02). The presence of *Chlamydia* antibodies is associated with a fourfold risk of ectopic pregnancy (OR 4.4; CI 1.70–11.48) (Table 3).

Table 4 shows that prior *Chlamydia trachomatis* infection, single women, and a history of previous pelvic/abdominal surgery are independently associated with ectopic pregnancy at Mbarara Regional Referral Hospital.

Logistic regression model analysis of risk factors for ectopic pregnancy showed a fourfold increase in the risk of ectopic pregnancy in those with *C. trachomatis* antibodies when controlling for previous history of pelvic inflammatory disease (OR 4.9; 95% CI 1.15–21.29).

Controlling for the effects of sociodemographic characteristics, previous history of pelvic inflammatory disease, marital status, and number of sexual partners showed that single women (OR 12.0; 95% CI 2.21–65.32) and having a history of previous abdominal/pelvic surgery (OR 6.9; 95% CI 1.47–32.98) were shown to be more likely to be associated with ectopic pregnancy as well.

## 4. Discussion

Serum immunoglobulin G antibodies have been associated with endosalpingeal damage and ectopic pregnancy resulting from pelvic inflammatory disease [8, 9]. In this study, the prevalence of chlamydial antibodies was significantly higher (60.0%) in patients with ectopic pregnancy than in women with intrauterine pregnancy (26.32%). This finding confirms the report of other researchers that patients with ectopic pregnancy are more likely to have immunoglobulin G antibodies against *C. trachomatis* when compared with women with intrauterine pregnancy [4, 9]. This suggested relationship was further strengthened by the finding in this study of a fourfold increase in the risk of ectopic pregnancy in women with chlamydial antibodies. The prevalence of prior *C. trachomatis* infection, as evidenced by the presence of chlamydial antibodies in this and other studies, underscores the reason why ectopic pregnancy has remained a major complication of reproductive health of women and why the incidence is increasing in parallel with the rise in the rate of chlamydial infection [10]. This study also found that women with ectopic pregnancy were more likely to be single, corroborating the findings in another Nigerian study [11]. This can be explained by the fact that single women are more likely to have multiple sexual partners than married women. In examining the risk factors for *Chlamydia* infection among patients with ectopic pregnancy and normal pregnancy, this study looked at the sexual and reproductive characteristics of both groups. The results of univariate analysis showed that women with ectopic pregnancy engaged in sexual intercourse at a significantly younger age than women with intrauterine pregnancy. This finding is in line with previous studies which linked early coitarche with *Chlamydia trachomatis* infection [12]. Adolescents are prone to sexually transmitted disease (STD) because of high risk sexual behaviour. Biologically, the adolescents are particularly at risk of STD because the columnar epithelium, which is susceptible to *Chlamydia* and gonococcal organisms, extends from the endocervical canal to the ectocervix making it fully exposed to pathogens. They also have difficulties using barrier methods of contraception and may have less access to STD care because of limited facilities, negative peer pressure, concealment, and restrictive policies especially in the developing countries [13]. Previous history of pelvic inflammatory disease was significantly higher in patients with ectopic pregnancy when compared with women with normal pregnant controls. This agrees with the findings of previous studies in this regard [8]. In contrast to other studies [8], this study found that induced abortions were relatively low in women with ectopic pregnancy and did not have any association with ectopic pregnancy. This could be explained by the small number of participants in this study who had a history of induced abortions, and therefore it was difficult to assess statistical difference with such a small number.

In our study, previous history of abdominal/pelvic surgery was associated with ectopic pregnancy and this compares with studies done in other areas [14]. This could be explained by the fact that previous surgery in the pelvic area or on the tubes can cause adhesions and impede the egg's movement causing implantation in the tubes and other sites.

However, when these were subjected to logistic regression model (multivariate) analysis, patients with *Chlamydia* antibodies showed a fourfold increase in the risk of ectopic pregnancy when controlling for previous history of pelvic inflammatory disease. In addition, the multivariate analysis revealed that women with a history of previous abdominal/pelvic surgery and those who were single were significantly associated with ectopic pregnancy.

## 5. Conclusion

This study has shown that a greater proportion of women with ectopic pregnancy had serological evidence of prior *C. trachomatis* infection than women with intrauterine pregnancy. It also demonstrated a fourfold risk association between prior *C. trachomatis* infection and ectopic pregnancy. Furthermore, being single and having a history of previous abdominal/pelvic surgery were positively associated with ectopic pregnancy. There is therefore a need for the sexual reproductive health division in the MoH to strengthen/improve sex education and for prompt and effective treatment of sexually transmitted infections.

## Figures and Tables

**Table 1:** Sociodemographic characteristics (*N* = 101).

Characteristic	Controls, *n* (%)	Cases, *n* (%)	*p* value
*Age in years*			
16–19	12 (15.79)	1 (4.00)	
20–24	38 (50.00)	6 (24.00)	
25–34	21 (27.63)	14 (56.00)	0.011
35–42	5 (6.58)	4 (16.00)	
Mean age	27.8 ± 4.3	25.2 ± 5.9	
*District*			
Mbarara	68 (90.67)	20 (80.00)	
Greater Bushenyi	4 (5.33)	4 (16.00)	0.234
Others	4 (4.00)	1 (4.00)	
*Residence type*			
Rural	38 (50.00)	13 (52.00)	0.862
Urban	38 (50.00)	12 (48.00)	
*Tribe*			
Banyankore	59 (77.63)	20 (80.00)	
Baganda	5 (6.58)	3 (12.00)	
Bakiga	7 (9.21)	2 (8.00)	0.496
Others	5 (6.58)	0 (0.00)	
*Level of education*			
No formal education	2 (2.63)	0 (0.00)	
Primary education	38 (50.00)	6 (24.00)	
Secondary education	24 (31.58)	11 (44.00)	0.075
Tertiary education	12 (15.79)	8 (32.00)	
*Marital status*			
Single	5 (6.58)	12 (48.00)	0.000
Married	68 (89.47)	12 (48.00)	
Separated	3 (3.95)	1 (4.00)	
*Religion*			
Christian	44 (57.89)	14 (56.00)	0.868
Moslem	32 (42.11)	11 (44.00)	
*Occupation*			
Unemployed	13 (17.33)	1 (4.00)	
Peasant	35 (46.67)	3 (12.00)	
Business	21 (28.00)	15 (60.00)	0.001
Professional	6 (8.00)	6 (24.00)	
*Income in Uganda (shs)*			
≤90,000	24 (31.58)	24 (96.00)	0.000
≥90,000	52 (68.42)	1 (4.00)	

**Table 2:** Sexual and reproductive characteristics (*N* = 101).

Characteristic	Controls, *n* (%)	Cases, *n* (%)	*p* value
*Parity*			
1	41 (53.95)	1 (4.00)	
2	22 (28.95)	21 (84.00)	0.000
>3	13 (17.11)	3 (12.00)	
*Menarche*			
<12 years	2 (2.67)	1 (4.00)	
12–14 years	37 (49.33)	21 (84.00)	0.006
>14 years	36 (48.00)	3 (12.00)	
*Coitarche*			
<19 years	46 (60.00)	25 (100.00)	0.000
>19 years	30 (40.00)	0 (0.00)	
*Multiple sexual partners*			
No	57 (75.00)	11 (44.00)	
Yes	19 (25.00)	14 (56.00)	0.004
*Induced abortion*			
None	74 (97.37)	20 (80.00)	
1–4	2 (2.63)	5 (20.00)	0.003
*History of previous PID*			
No	68 (89.47)	1 (4.00)	
Yes	8 (10.53)	24 (96.00)	0.002
*History of puerperal/postabortal sepsis*			
No	75 (98.68)	24 (96.00)	
Yes	1 (1.32)	1 (4.00)	0.403
*History of abdominal/pelvic surgery*			
No	70 (92.11)	12 (48.00)	
Yes	6 (7.89)	13 (52.00)	0.000
*History of IUCD usage*			
No	73 (97.33)	24 (96.00)	
Yes	2 (2.67)	1 (4.00)	0.735

**Table 3:** Confirmed *Chlamydia trachomatis* IgG antibodies in both controls and cases.

*Chlamydia* IgG antibodies	Control, *n* (%)	Cases, *n* (%)	*p* value
Negative	56 (73.67)	10 (40)	
Positive	20 (26.32)	15 (60)	**0.002**

**Table 4:** Factors associated with ectopic pregnancy at multivariate analysis.

Variable	Univariable		Multivariable	
	OR (95% CI)	*p* value	aOR (95% CI)	*p* value
*Chlamydia IgG antibodies*				
No	1.0		1.0	
Yes	4.4 (1.70–11.48)	**0.0019**	4.9 (1.15–21.29)	**0.0019**
*Marital status*				
Single	13.6 (4.05–45.62)	**0.0000**	12.0 (2.21–65.32)	**0.0026**
Married	1.0		1.0	
Separated	1.8 (0.18–19.70)		0.6 (0.04–8.48)	
*Parity*				
1	1.0		1.0	
2	39.1 (4.92–310.73)		24.9 (1.47–205.98)	
>3	9.4 (0.90–98.96)	**0.0000**	6.9 (1.47–32.98)	0.3752
*Menarche*				
<12 years	1.0		1.0	
12–14 years	1.1 (0.09–13.27)	**0.0031**	3.4 (0.77–14.82)	0.2796
>14 years	0.1 (0.01–2.41)		1.1 (0.11–11.50)	
*Coitarche*				
<19 years	0.6 (0.02–0.34)	**0.0000**	0.9 (0.37–2.28)	0.8622
>19 years	1.0		1.0	
*Multiple sexual partners*				
No	1.0		1.0	
Yes	3.8 (1.48–9.82)	**0.0051**	3.1 (0.18–11.88)	0.4376
*Induced abortion*				
None	1.0		1.0	
1–4	9.2 (1.66–51.27)	**0.0067**	7.0 (0.43–46.68)	0.8681
*History of abdominal/pelvic surgery*				
No	1.0		1.0	
Yes	12.6 (4.02–39.71)	**0.0001**	6.9 (1.47–32.98)	**0.0000**
*History of previous PID*				
No	1.0		1.0	
Yes	104 (24.23–171.5)	**0.0000**	85 (33.14–169.4)	0.5689
